# Cortisol as a Predictor of Nocturnal Hypoglycemia in Insulin-Treated Diabetes: A Cross-Sectional Study

**DOI:** 10.7759/cureus.89706

**Published:** 2025-08-09

**Authors:** Tetsushi Nasu, Eri Tamagawa, Atsuyo Fujita

**Affiliations:** 1 Diabetes Medicine, Hannan Municipal Hospital, Hannan, JPN

**Keywords:** advanced type 2 diabetes, asymptomatic hypoglycemia, cortisol, insulin-treated diabetes, nocturnal hypoglycemia, type 1 diabetes

## Abstract

Introduction: Nocturnal hypoglycemia (NH) is a major clinical concern in insulin-treated diabetes due to blunted autonomic responses and reduced awareness of hypoglycemia during sleep. We investigated the association between NH and early morning fasting cortisol levels in this population.

Methods: This case-control study included 30 insulin-treated adults with type 1 diabetes (n = 22) or advanced type 2 diabetes (n = 8) and depleted endogenous insulin secretion. Glucose profiles were assessed using intermittently scanned continuous glucose monitoring. NH was defined as glucose levels <70 mg/dL between 00:00 and 06:00. Fasting-morning serum cortisol, plasma glucagon, and serum C-peptide levels were also measured. The clinical and biochemical parameters were compared between patients with and without NH.

Results: NH occurred in 15 of 30 patients (50.0%), including three (10.0%) with level 2 hypoglycemia (<54 mg/dL). There were no significant differences between the groups in terms of age, diabetes type, disease duration, HbA1c level, body mass index (BMI), insulin dose, or C-peptide level. However, fasting cortisol levels were significantly lower in patients with NH. Logistic regression analysis showed that lower cortisol levels were independently associated with NH (odds ratio: 0.708; 95% confidence interval: 0.52-0.97; p < 0.05). Receiver operating characteristic curve analysis identified a cortisol threshold of 10.7 μg/dL for predicting NH (area under the curve = 0.79, sensitivity = 0.73, specificity = 0.80).

Conclusions: Lower early morning cortisol levels may serve as an independent risk factor for NH in patients with insulin-treated diabetes. Cortisol measurements may help identify individuals at risk of asymptomatic NH.

## Introduction

Type 1 and advanced type 2 diabetes are associated with hyperglycemia resulting from reduced insulin secretion by the pancreas. Insulin is required to regulate blood glucose levels. The Diabetes Control and Complications Trial and Epidemiology of Diabetes Interventions and Complications underscore the importance of maintaining tight glycemic control in patients with type 1 diabetes [1-3]. However, achieving optimal control in insulin-dependent states remains challenging owing to the wide fluctuations in glucose levels, including hypoglycemia and hyperglycemia. As blood glucose decreases during treatment, counterregulatory hormones, such as catecholamines, cortisol, growth hormones, and glucagon, are released [4-6]. During the day, patients are typically aware of hypoglycemic symptoms and consume oral glucose to prevent severe hypoglycemia. However, nocturnal hypoglycemia (NH) occurs when glucose levels decrease during sleep, and patients may remain unaware of the symptoms, delaying corrective action. Severe hypoglycemia can result in cardiac events and altered consciousness [7-12]. Even in the absence of severe hypoglycemia, cognitive function and mood may deteriorate the following day, fatigue may increase, and the quality of life may decline. Autonomic responses to hypoglycemia are further diminished during sleep in individuals with type 1 diabetes. These individuals are considerably less likely to awaken during hypoglycemia, potentially because of blunted sympathoadrenal responses and sleep-associated hypoglycemia-related autonomic failure [13-15]. Therefore, it is essential to monitor nighttime blood glucose levels and implement strategies to prevent hypoglycemia in patients with insulin-dependent diabetes. In this study, we continuously monitored the overnight glucose levels and examined the relationship between NH onset and baseline fasting cortisol, glucagon, and insulin secretion.

## Materials and methods

This case-control study was conducted at our hospital from April 2024 to March 2025. We included patients with type 1 and advanced type 2 diabetes who had previously undergone evaluation of endogenous insulin secretion and were suspected to be insulin dependent based on fasting C-peptide (<0.6 ng/mL) or urinary C-peptide (<20 μg/day) levels. All participants were adults aged ≥20 years and were managed with insulin therapy. Patients receiving chemotherapy or steroid therapy, and those with an estimated glomerular filtration rate <30 mL/min/1.73 m2 or gestational diabetes mellitus, were excluded. In addition, individuals using a sensor-augmented pump or a sensor-augmented pump with hybrid closed-loop functionality were not eligible for inclusion. Given the exploratory nature of this study, all available cases were analyzed, and no prior sample size calculation was performed.

Glucose monitoring

Blood glucose levels were monitored using an intermittently scanned continuous glucose monitoring system (isCGM) (FreeStyle Libre; Abbott Diabetes Care, Alameda, CA, USA). The device measures interstitial glucose via a sensor inserted in the subcutaneous tissue of the upper arm, providing glucose readings at 15-minute intervals. Data from the 14 days preceding the visit were analyzed to evaluate the association between glucose variability and hormones such as endogenous insulin, cortisol, and glucagon. For the analysis, data from the first and last days of sensor wear were excluded to account for measurement instability. The focus was on the isCGM data collected between 00:00 and 06:00. According to the International Hypoglycemia Study Group, level 1 NH is defined as glucose <70 mg/dL and level 2 NH as glucose <54 mg/dL [16-18]. We specifically analyzed the occurrence of level 1 NH events during this period.

Sample collection

Blood samples were collected after overnight fasting for at least eight hours. Fasting serum cortisol and C-peptide levels were measured by SRL Inc. (Tokyo, Japan). Serum cortisol concentration was determined using an electrochemiluminescence immunoassay (ECLIA) with a commercially available kit (Elecsys Cortisol II; Roche Diagnostics K.K., Tokyo, Japan). The detection limit was 0.054 μg/dL, and the intra-assay coefficient of variation (CV) was <15%. Serum C-peptide levels were measured using a commercially available chemiluminescent enzyme immunoassay (CLEIA) kit (Lumipulse C-peptide; Fujirebio Inc., Tokyo, Japan). Values below the quantification limit were considered zero for the statistical analysis.

Statistical analysis

Data are presented as the median (interquartile range). Between-group differences were evaluated using the Mann-Whitney U test. Categorical variables were compared using Fisher’s exact test. These analyses, along with multiple logistic regressions, were performed using EZR version 1.64 (Saitama Medical Center, Jichi Medical University, Saitama, Japan), a graphical user interface for R (R Foundation for Statistical Computing, Vienna, Austria) [19]. EZR is a modified version of R Commander, with functions commonly used in biostatistics.

## Results

Forty patients were initially enrolled in the study; however, 10 were excluded. Of these, five were excluded because reassessment of insulin secretory capacity did not confirm insulin dependence. Two patients had a history of pancreatic resection, two were undergoing chemotherapy, and one was excluded because of transfer to another hospital after enrollment.

Table 1 shows the general characteristics of the 30 patients included in the analysis. Twenty-two patients had type 1 diabetes, and the remaining eight had type 2 diabetes. All patients had a disease duration of at least six months, with a median duration of 16.5 years and a median age of 68.0 years. Regarding treatment, 26 patients received intensive insulin therapy, three received continuous subcutaneous insulin infusion, and one was treated with long-acting insulin alone. None of the patients underwent the hybrid closed-loop therapy. During the isCGM use period, none of the patients experienced severe hypoglycemia during the day or night, nor did they develop any comorbidities. According to the 14-day isCGM analysis, 15 of the 30 patients exhibited nocturnal hypoglycemic unawareness. The criteria for NH were defined as glucose levels of 70 mg/dL for level 1 hypoglycemia and <54 mg/dL for level 2 hypoglycemia between 0:00 and 6:00 a.m., with a frequency of ≥1% of total readings. The CGM results showed that 15 patients (50.0%) had level 1 NH and three patients (10.0%) had level 2 hypoglycemia. In this high-risk group of individuals with type 1 diabetes, the adjustment of insulin therapy is often challenging, and the risk of hypoglycemia, especially nocturnal hypoglycemia, is elevated. The patients in this study were of advanced age. To detect nocturnal hypoglycemia unawareness, the focus was on the presence or absence of blood glucose levels <70 mg/dL (level 1). Table 1 categorizes the patients into groups with and without hypoglycemic events. No differences were found between the groups in terms of type 1 or type 2 diabetes, sex, age, disease duration, or body mass index (BMI). Additionally, glycated hemoglobin (HbA1c) levels were comparable, with no notable differences in basal insulin dosage. Fasting C-peptide, glucagon, and cortisol levels were analyzed according to the NH susceptibility. Although C-peptide and glucagon levels did not significantly differ with respect to NH susceptibility, a significant difference was observed in basal cortisol levels.

**Table 1:** Comparison of patient backgrounds between groups with and without nocturnal hypoglycemia Categorical data are presented as the number (%) of patients and were compared using Fisher’s exact test. Continuous variables are presented as medians (interquartile ranges) and were compared using the Mann–Whitney U test. Abbreviations: HbA1c, glycated hemoglobin; eGFR, estimated glomerular filtration rate; ACTH, adrenocorticotropic hormone, GH: growth hormone

Variables	Overall (n = 30)	Nocturnal Hypoglycemia Group (n = 15)	No Nocturnal Hypoglycemia Group (n = 15)	U-value	p-value
Diabetes Type					
Type 1 Diabetes, n (%)	22(73.3)	12(80.0)	10(66.7)	-	0.682
Type 2 Diabetes, n (%)	8(26.7)	3(20.0)	5(33.3)		
Sex					
Male, n (%)	14(46.7)	8(53.3)	6(40.0)	-	0.715
Female, n (%)	16(53.3)	7(46.7)	9(60.0)		
Age (years)	68.0(48.0, 78.0)	62.0(46.5, 76.5)	71.0(57.0, 80.0)	25.0	0.177
Duration of diabetes (years)	16.5(6.25, 22.5)	16.0(6.00, 20.0)	19.0(6.50, 27.0)	3.5	0.648
Body mass index (kg/m^2^)	21.6(19.8, 24.3)	23.5(21.0, 24.8)	20.1(19.6, 22.2)	43.0	0.093
HbA1c (%)	7.70(7.32, 8.28)	7.40(7.00, 8.75)	7.90(7.50, 8.15)	0.5	0.771
Plasma glucose (mg/dL)	167(120, 190)	168(118, 188)	165(125, 187)	4.5	0.901
C-peptide (ng/mL)	0.11(0.00, 0.33)	0.14(0.03, 0.27)	0.06(0.00, 0.36)	19.5	0.612
Glucagon (pg/mL)	13.5(11.4, 18.3)	13.0(10.1, 21.1)	14.1(12.8, 17.7)	6.0	0.575
Cortisol (μg/dL)	11.1(9.1, 14.8)	9.5(8.3, 11.4)	13.7(11.1, 15.9)	58.5	<0.05
ACTH (pg/mL)	26.4(20.4, 34.8)	32.7(26.6, 36.0)	20.8(18.7, 32.1)	52.0	0.100
GH (ng/mL)	2.22(1.15, 3.26)	1.55(0.97, 3.28)	2.56(1.46, 3.15)	20.0	0.411
Noradrenaline (pg/mL)	570.5(431.3, 655.3)	583.0(450.0, 741.5)	570.5(421.5, 625.0)	41.5	0.777
Adrenaline (pg/mL)	43.0(28.0, 58.0)	48.5(37.8, 60.5)	40.5(26.3, 54.0)	49.0	0.504
Dopamine (pg/mL)	16.0(11.0, 22.0)	19.0(12.5, 22.5)	16.0(6.5, 27.0)	48.5	0.763
eGFR (mL/min/1.73 m^2^)	77.7(64.3, 87.2)	77.8(70.1, 98.0)	76.9(61.1, 85.1)	27.0	0.419
Dose of basal insulin (units/kg/day)	0.16(0.12, 0.22)	0.17(0.13, 0.21)	0.16(0.11, 0.23)	10.0	0.917
Dose of bolus insulin (units/kg/day)	0.33(0.25, 0.46)	0.33(0.26, 0.54)	0.33(0.26, 0.41)	20.0	0.604

Table 2 presents CGM metrics for the two groups. No significant differences were found in average glucose levels or CV during nighttime or daytime. As expected, time below range (TBR) during nighttime differed between groups due to the hypoglycemia definition. Notably, a significant difference was also observed in daytime TBR. Table 3 shows the logistic regression analysis identifying factors influencing NH occurrence. NH presence or absence was the dependent variable. The analysis indicated that lower cortisol levels were significantly associated with increased NH risk (odds ratio = 0.708, 95% confidence interval: 0.52-0.97, p < 0.05). No significant associations were observed for other factors, including HbA1c, C-peptide, BMI, or basal insulin dose.

**Table 2:** Comparison of clinical data and continuous glucose monitoring metrics between groups with and without nocturnal hypoglycemia Continuous variables are presented as medians (interquartile ranges) and were compared using the Mann–Whitney U test. Statistical significance was set at p < 0.05. Abbreviations: CV, coefficient of variation; TBR, time below range; TIR, time in range; TAR, time above range.

Variables	Nocturnal Hypoglycemia Group (n = 15)	No Nocturnal Hypoglycemia Group (n = 15)	U-value	p-value
Nighttime (24.00–06.00 hours)				
Average glucose (mg/dL)	173(131, 188)	159(141, 203)	2	0.694
CV (%)	34.3(29.5, 399.9)	27.3(23.0, 35.9)	51	0.071
TBR >1% (N)	15	0		
TBR (%)	3.2(2.4, 4.8)	0.0(0.0, 0.2)	0	<0.05
TIR (%)	55.3(41.7, 81.3)	71.3(40.5, 77.4)	10	0.917
TAR (%)	39.9(14.9, 51.3)	28.7(22.3, 59.5)	3	0.663
Daytime (06.00–24.00 hours)				
Average glucose (mg/dL)	166(146, 185)	182(166, 192)	18	0.290
CV(%)	36.5(32.2, 46.1)	31.8(28.2, 38.7)	53	0.059
TBR >1% (N)	9	3		
TBR (%)	1.1(0.7, 2.7)	0.0(0.0, 0.2)	48	<0.05
TIR (%)	59.8(46.5, 70.6)	54.3(49.0, 64.0)	14	0.787
TAR (%)	34.1(24.6, 48.2)	44.6(33.9, 50.3)	14	0.373

**Table 3:** Logistic regression analysis for nocturnal hypoglycemia Abbreviations: HbA1c, glycated hemoglobin

Factor	Odds Ratio (OR)	95% Confidence Interval (CI)	p-value
HbA1c (%)	0.896	0.31–2.57	0.838
C-peptide (ng/mL)	2.290	0.35–15.00	0.388
Cortisol (μg/dL)	0.708	0.52–0.97	0.031
Body mass index (kg/m^2^)	1.070	0.75–1.51	0.724
Dose of basal insulin (units/kg/day × 10)	0.968	0.37–2.51	0.946

**Figure 1:**
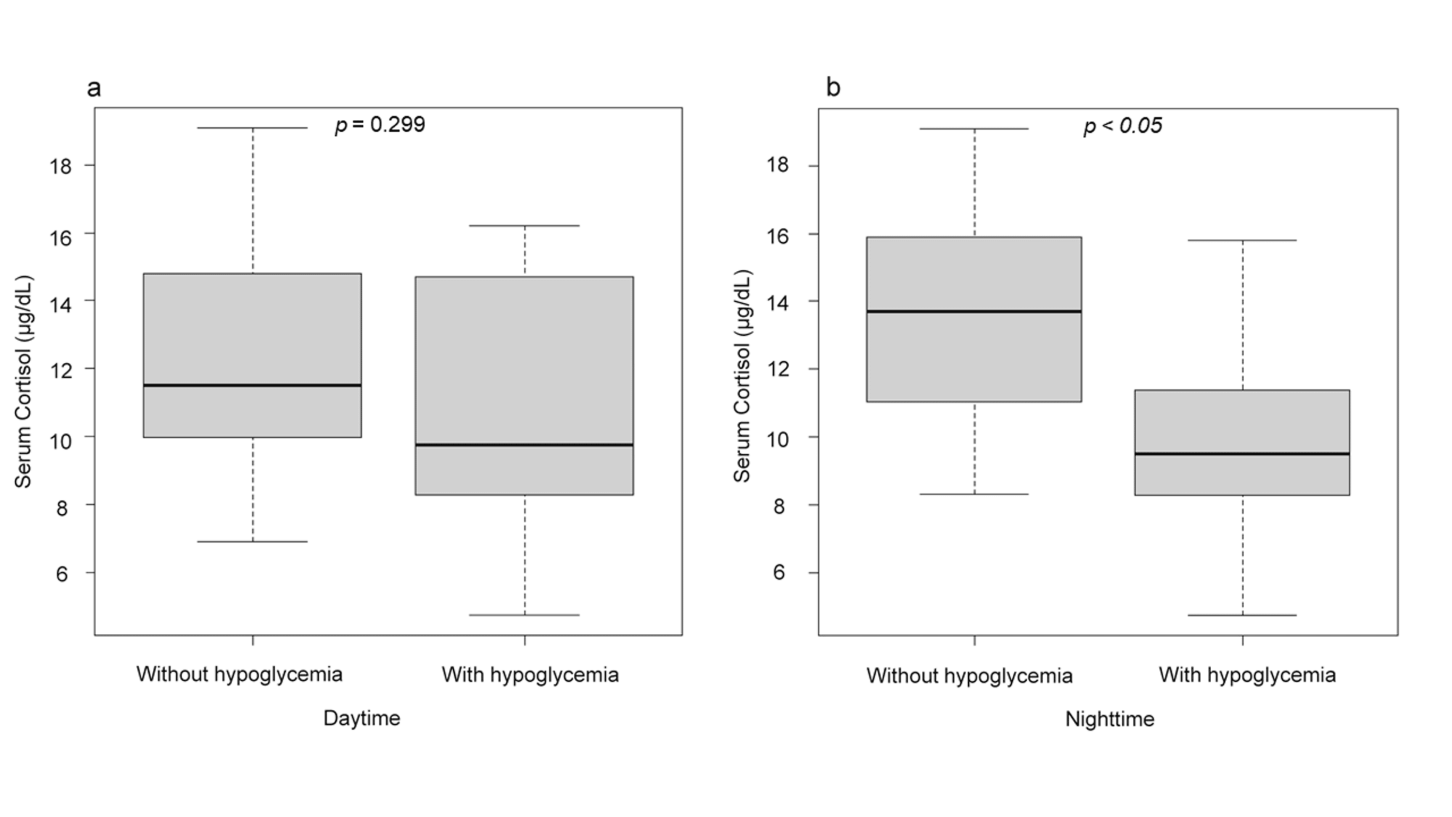
Comparison of fasting cortisol levels in patients with or without nocturnal and daytime hypoglycemia Boxplots of cortisol concentrations measured in patients with or without hypoglycemia (blood glucose < 70 mg/dL). The lower and upper fences represent the 25th and 75th percentiles, respectively, with the median indicated between them. Statistical analysis was performed using the Mann-Whitney U test. a: Daytime (06:00–24:00); b: Nighttime (00:00–06:00).

**Figure 2:**
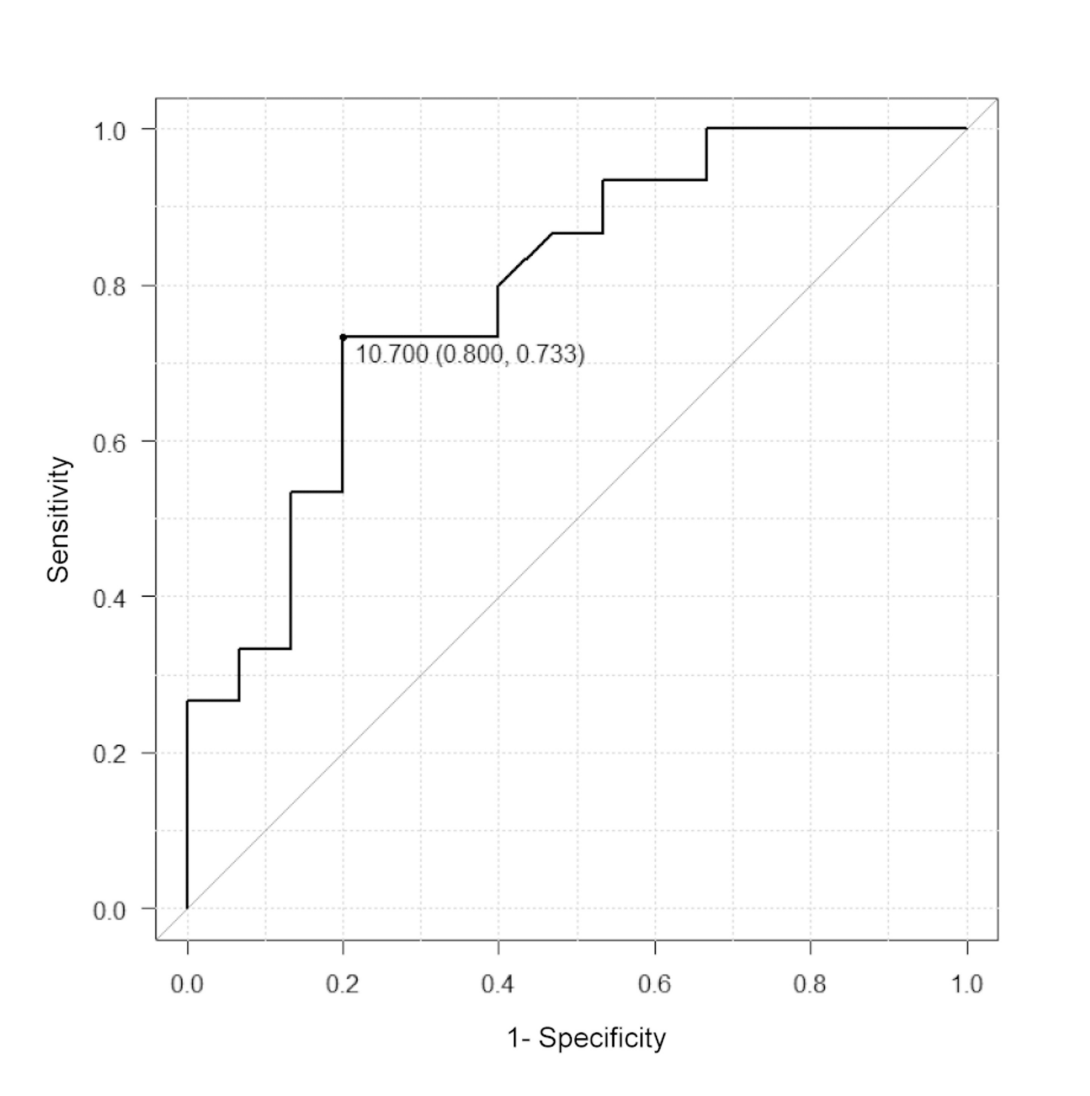
ROC curve of fasting serum cortisol levels stratified by nocturnal hypoglycemia Receiver operating characteristic (ROC) curve analysis for predicting nocturnal hypoglycemia (NH). The optimal cut-off value for basal cortisol was 10.7 μg/dL (sensitivity = 0.73, specificity = 0.80, area under the curve = 0.79, p < 0.05).

Figure 1 illustrates the association between hypoglycemia presence and basal cortisol levels. Figure 1A shows a box-and-whisker plot of cortisol levels for daytime hypoglycemia, while Figure 1B presents cortisol levels for nighttime hypoglycemia. In Figure 1B, baseline serum cortisol levels differed significantly between groups with and without NH. Conversely, Figure 1A shows no significant difference in baseline cortisol levels between groups classified by the presence or absence of daytime hypoglycemia. Figure 2 shows the receiver operating characteristic (ROC) curve for baseline cortisol levels. The optimal cut-off value was 10.7 μg/dL, with a sensitivity of 0.73, specificity of 0.80, and area under the curve of 0.79 (p < 0.05). 

## Discussion

Type 1 diabetes is characterized by autoimmune destruction of the pancreatic beta cells, resulting in insulin deficiency. Consequently, insulin therapy is essential, with most patients relying on multiple daily injections or continuous subcutaneous insulin infusions to maintain their blood glucose levels. In this context, counter-regulatory hormones, such as glucagon, cortisol, and epinephrine, prevent and correct hypoglycemia. Understanding the roles of these hormones is particularly important for avoiding hypoglycemia during tight glycemic control, which remains a significant challenge in insulin-dependent diabetes.

Long-standing diabetes impairs the secretion of counter-regulatory hormones, including catecholamines and cortisol, during physiological responses to hypoglycemia. This diminished hormonal response contributes to the development of hypoglycemia unawareness and increases the risk of severe NH [14,19,20]. However, few studies have evaluated insulin secretion and glucagon or cortisol levels in relation to NH in patients with insulin-dependent diabetes by using CGM.

To our knowledge, this study is the first to examine the relationship between morning fasting cortisol levels and NH detected using CGM in patients with type 1 and advanced type 2 diabetes with insulin dependence. Furthermore, we explored the possibility that the cortisol response to hypoglycemia, a key component of the counter-regulatory system, may be impaired in these individuals.

The CGM data from this study showed that approximately half of the participants experienced level 1 NH (<70 mg/dL), whereas a smaller subset experienced level 2 events. In this high-risk population, adjusting insulin doses is often challenging, and the risk of NH remains high. Given the advanced age of the participants, we focused on asymptomatic NH by identifying those with nocturnal glucose levels <70 mg/dL. Among the various biochemical parameters analyzed, only early morning cortisol levels were significantly associated with NH. Logistic regression analyses of HbA1c, C-peptide, and basal insulin doses revealed distinct differences, suggesting a potential link between adrenal insufficiency (AI) and autonomic neuropathy.

AI is typically characterized by nonspecific symptoms, including general fatigue, appetite loss, weight loss, gastrointestinal symptoms, hypotension, mental disorders, arthralgia, and hypoglycemia [21-24]. However, diagnosis is often delayed or missed in clinical practice due to the subtle or absent nature of symptoms, particularly in the early or subclinical stages. Routine laboratory findings, such as hyponatremia, hypoglycemia, normocytic anemia, hypocholesterolemia, eosinophilia, and occasionally leukocytosis, may provide indirect clues [25-27]. In this study, none of the participants exhibited overt clinical symptoms of adrenal insufficiency, and none had a basal serum cortisol concentration <4 μg/dL. Nevertheless, most individuals (n = 29, 96.7%) presented early morning cortisol levels <18 μg/dL, a threshold suggestive of possible AI.

Although adrenocorticotropic hormone (ACTH) stimulation testing could not be performed in this outpatient setting, ROC analysis demonstrated that a basal cortisol level <10.7 μg/dL was significantly associated with NH (sensitivity = 0.73, specificity = 0.80, area under the curve = 0.79, p < 0.05). This finding suggests a potential physiological link between subclinical adrenal hypofunction and impaired counter-regulatory responses to NH. Even in the absence of overt AI symptoms, blunted cortisol responses may increase susceptibility to nocturnal glucose decline, particularly in insulin-dependent patients with reduced endogenous insulin secretion and altered hypothalamic-pituitary-adrenal (HPA) axis dynamics. Further studies employing dynamic hormonal tests, such as low-dose ACTH stimulation or insulin-induced hypoglycemia, are required to confirm the clinical relevance of latent AI in glucose regulation.

Given the ease of measuring early morning basal cortisol levels, this measurement may serve as a practical screening tool for high-risk populations to prevent hypoglycemia-related adverse outcomes. Before the widespread use of CGM, asymptomatic NH was often undetected because episodes without overt symptoms were frequently overlooked. Recent studies, including those by Kanazawa et al., examined the association between CGM-detected NH and latent adrenal insufficiency, particularly in patients with type 2 diabetes [28]. Although their study population differed from ours, which included only insulin-dependent individuals with type 1 or advanced type 2 diabetes, their findings support the concept that underlying adrenal dysfunction may contribute to the risk of NH. Our findings suggest that measuring early morning fasting cortisol levels could serve as a simple screening method to identify patients at an increased risk of NH. While an ideal assessment of adrenal function requires corticotropin stimulation testing to detect latent adrenal insufficiency, this study was limited to outpatient measurements of early morning basal cortisol levels. This study has some limitations. We acknowledge that the relatively small sample size may limit the statistical power to detect certain associations and could widen confidence intervals, leading to greater uncertainty in the estimates. Therefore, the results should be interpreted with caution and considered as indicative rather than definitive. Future studies with larger sample sizes or multi-center designs are warranted to validate these findings. Moreover, a single early morning cortisol measurement is insufficient to evaluate diurnal hormonal variations or fully assess adrenal responsiveness. Future studies should incorporate dynamic hormone testing to examine the correlation between diurnal cortisol patterns and CGM data.

## Conclusions

In conclusion, among insulin-dependent patients with diminished insulin secretion capacity, focusing on daytime glycemic variability detected by CGM, asymptomatic NH, along with early morning cortisol screening, may improve prevention strategies. These findings underscore the importance of considering adrenal function in the comprehensive management of hypoglycemic risk in patients with diabetes.

## References

[1] Nathan DM, Genuth S, Lachin J (1993). The effect of intensive treatment of diabetes on the development and progression of long-term complications in insulin-dependent diabetes mellitus. N Engl J Med.

[2] (2016). Intensive diabetes treatment and cardiovascular outcomes in type 1 diabetes: the DCCT/EDIC study 30-year follow-up. Diabetes Care.

[3] Nathan DM, Cleary PA, Backlund JY (2005). Intensive diabetes treatment and cardiovascular disease in patients with type 1 diabetes. N Engl J Med.

[4] Cryer PE, Davis SN, Shamoon H (2003). Hypoglycemia in diabetes. Diabetes Care.

[5] Cryer PE (2006). Mechanisms of sympathoadrenal failure and hypoglycemia in diabetes. J Clin Invest.

[6] Rickels MR (2019). Hypoglycemia-associated autonomic failure, counterregulatory responses, and therapeutic options in type 1 diabetes. Ann N Y Acad Sci.

[7] Yaffe K, Falvey CM, Hamilton N (2013). Association between hypoglycemia and dementia in a biracial cohort of older adults with diabetes mellitus. JAMA Intern Med.

[8] Chin SO, Rhee SY, Chon S (2016). Hypoglycemia is associated with dementia in elderly patients with type 2 diabetes mellitus: an analysis based on the Korea National Diabetes Program cohort. Diabetes Res Clin Pract.

[9] Warren RE, Frier BM (2005). Hypoglycaemia and cognitive function. Diabetes Obes Metab.

[10] Hsu PF, Sung SH, Cheng HM (2013). Association of clinical symptomatic hypoglycemia with cardiovascular events and total mortality in type 2 diabetes: a nationwide population-based study. Diabetes Care.

[11] Allen KV, Frier BM (2003). Nocturnal hypoglycemia: clinical manifestations and therapeutic strategies toward prevention. Endocr Pract.

[12] Henriksen MM, Andersen HU, Thorsteinsson B, Pedersen-Bjergaard U (2021). Effects of continuous glucose monitor-recorded nocturnal hypoglycaemia on quality of life and mood during daily life in type 1 diabetes. Diabetologia.

[13] Banarer S, Cryer PE (2003). Sleep-related hypoglycemia-associated autonomic failure in type 1 diabetes: reduced awakening from sleep during hypoglycemia. Diabetes.

[14] Cryer PE (2005). Mechanisms of hypoglycemia-associated autonomic failure and its component syndromes in diabetes. Diabetes.

[15] Danne T, Nimri R, Battelino T (2017). International consensus on use of continuous glucose monitoring. Diabetes Care.

[16] (2017). Glucose concentrations of less than 3.0 mmol/L (54 mg/dL) should be reported in clinical trials: a joint position statement of the American Diabetes Association and the European Association for the Study of Diabetes. Diabetes Care.

[17] Battelino T, Danne T, Bergenstal RM (2019). Clinical targets for continuous glucose monitoring data interpretation: recommendations from the international consensus on time in range. Diabetes Care.

[18] Kanda Y (2013). Investigation of the freely available easy-to-use software 'EZR' for medical statistics. Bone Marrow Transplant.

[19] Fabricius TW, Verhulst CE, Kristensen PL (2024). Counterregulatory hormone and symptom responses to hypoglycaemia in people with type 1 diabetes, insulin-treated type 2 diabetes or without diabetes: the Hypo-RESOLVE hypoglycaemic clamp study. Acta Diabetol.

[20] Fredheim S, Foli-Andersen P, Laerkholm G (2018). Adrenaline and cortisol levels are lower during nighttime than daytime hypoglycaemia in children with type 1 diabetes. Acta Paediatr.

[21] Yanase T, Tajima T, Katabami T (2016). Diagnosis and treatment of adrenal insufficiency including adrenal crisis: a Japan Endocrine Society clinical practice guideline [Opinion]. Endocr J.

[22] Husebye ES, Allolio B, Arlt W (2014). Consensus statement on the diagnosis, treatment and follow-up of patients with primary adrenal insufficiency. J Intern Med.

[23] Bornstein SR, Allolio B, Arlt W (2016). Diagnosis and treatment of primary adrenal insufficiency: an Endocrine Society Clinical Practice Guideline. J Clin Endocrinol Metab.

[24] Ten S, New M, Maclaren N (2001). Clinical review 130: Addison's disease 2001. J Clin Endocrinol Metab.

[25] Younes N, Bourdeau I, Lacroix A (2021). Latent adrenal insufficiency: from concept to diagnosis. Front Endocrinol (Lausanne).

[26] Yamamoto T (2018). Latent adrenal insufficiency: concept, clues to detection, and diagnosis. Endocr Pract.

[27] Bleicken B, Hahner S, Ventz M, Quinkler M (2010). Delayed diagnosis of adrenal insufficiency is common: a cross-sectional study in 216 patients. Am J Med Sci.

[28] Kanazawa K, Hijikata M, Kuwabara K (2025). Evaluating latent adrenal insufficiency in elderly patients with nocturnal hypoglycemia: a retrospective observational study. Endocr Pract.

